# Predictive value of plasma proenkephalin and neutrophil gelatinase-associated lipocalin in acute kidney injury and mortality in cardiogenic shock

**DOI:** 10.1186/s13613-021-00814-8

**Published:** 2021-02-05

**Authors:** Toni Jäntti, Tuukka Tarvasmäki, Veli-Pekka Harjola, Kari Pulkki, Heidi Turkia, Tuija Sabell, Heli Tolppanen, Raija Jurkko, Mari Hongisto, Anu Kataja, Alessandro Sionis, Jose Silva-Cardoso, Marek Banaszewski, Salvatore DiSomma, Alexandre Mebazaa, Mikko Haapio, Johan Lassus, for the CardShock investigators, Veli-Pekka Harjola, Marek Banaszewski, Lars Køber, Johan Lassus, Alexandre Mebazaa, Marco Metra, John Parissis, Jose Silva-Cardoso, Alessandro Sionis, Salvatore Di Somma, Jindrich Spinar, Katerina Koniari, Astrinos Voumvourakis, Apostolos Karavidas, Jordi Sans-Rosello, Montserrat Vila, Albert Duran-Cambra, Michela Bulgari, Valentina Lazzarini, Jiri Parenica, Roman Stipal, Ondrej Ludka, Marie Palsuva, Eva Ganovska, Petr Kubena, Matias G. Lindholm, Christian Hassager, Tom Bäcklund, Raija Jurkko, Kristiina Järvinen, Tuomo Nieminen, Kari Pulkki, Leena Soininen, Reijo Sund, Ilkka Tierala, Jukka Tolonen, Marjut Varpula, Tuomas Korva, Anne Pitkälä, Rossella Marino, Alexandra Sousa, Carla Sousa, Mariana Paiva, Inês Rangel, Rui Almeida, Teresa Pinho, Maria Júlia Maciel, Janina Stepinska, Anna Skrobisz, Piotr Góral

**Affiliations:** 1 Department of Cardiology, Heart and Lung Center, Helsinki University Hospital, University of Helsinki, 00029 HUS Helsinki, Finland; 2 Emergency Medicine, Department of Emergency Medicine and Services, Helsinki University Hospital, University of Helsinki, Helsinki, Finland; 3 HUSLAB Diagnostic Services, Helsinki University Hospital and University of Helsinki, Helsinki, Finland; 4 Internal Medicine, Department of Internal Medicine and Rehabilitation, Helsinki University Hospital, University of Helsinki, Helsinki, Finland; 5 Intensive Cardiac Care Unit, Cardiology Department, Hospital de La Santa Creu I Sant Pau, Biomedical Research Institute IIB‐SantPau, Universidad Autónoma de Barcelona, Barcelona, Spain; 6 CINTESIS, Department of Cardiology, São João Hospital Center, and Porto Medical School, University of Porto, Porto, Portugal; 7 Intensive Cardiac Therapy Clinic, National Institute of Cardiology, Warsaw, Poland; 8 Department of Medical Sciences and Translational Medicine, Sant’Andrea Hospital, University of Rome Sapienza, Rome, Italy; 9 INSERM U942, Department of Anesthesia and Critical Care, Hôpital Lariboisière, APHP, University Paris Diderot, Paris, France; 10 Nephrology, Department of Nephrology, Abdominal Center, Helsinki University Hospital, University of Helsinki, Helsinki, Finland

**Keywords:** Cardiogenic shock, Acute kidney injury, AKI, Mortality, Prognosis, Proenkephalin, PENK, NGAL

## Abstract

**Background:**

Acute kidney injury (AKI) is a frequent form of organ injury in cardiogenic shock. However, data on AKI markers such as plasma proenkephalin (P-PENK) and neutrophil gelatinase-associated lipocalin (P-NGAL) in cardiogenic shock populations are lacking. The objective of this study was to assess the ability of P-PENK and P-NGAL to predict acute kidney injury and mortality in cardiogenic shock.

**Results:**

P-PENK and P-NGAL were measured at different time points between baseline and 48 h in 154 patients from the prospective CardShock study. The outcomes assessed were AKI defined by an increase in creatinine within 48 h and all-cause 90-day mortality. Mean age was 66 years and 26% were women. Baseline levels of P-PENK and P-NGAL (median [interquartile range]) were 99 (71–150) pmol/mL and 138 (84–214) ng/mL. P-PENK > 84.8 pmol/mL and P-NGAL > 104 ng/mL at baseline were identified as optimal cut-offs for AKI prediction and independently associated with AKI (adjusted HRs 2.2 [95% CI 1.1–4.4, *p* = 0.03] and 2.8 [95% CI 1.2–6.5, *p* = 0.01], respectively). P-PENK and P-NGAL levels at baseline were also associated with 90-day mortality. For patients with oliguria < 0.5 mL/kg/h for > 6 h before study enrollment, 90-day mortality differed significantly between patients with low and high P-PENK/P-NGAL at baseline (5% vs. 68%, *p* < 0.001). However, the biomarkers provided best discrimination for mortality when measured at 24 h. Identified cut-offs of P-PENK_24h_ > 105.7 pmol/L and P-NGAL_24h_ > 151 ng/mL had unadjusted hazard ratios of 5.6 (95% CI 3.1–10.7, *p* < 0.001) and 5.2 (95% CI 2.8–9.8, *p* < 0.001) for 90-day mortality. The association remained significant despite adjustments with AKI and two risk scores for mortality in cardiogenic shock.

**Conclusions:**

High levels of P-PENK and P-NGAL at baseline were independently associated with AKI in cardiogenic shock patients. Furthermore, oliguria before study inclusion was associated with worse outcomes only if combined with high baseline levels of P-PENK or P-NGAL. High levels of both P-PENK and P-NGAL at 24 h were found to be strong and independent predictors of 90-day mortality.

*Trial registration*: NCT01374867 at www.clinicaltrials.gov, registered 16 Jun 2011—retrospectively registered

## Background

Cardiogenic shock is a severe state of hypoperfusion, caused by low cardiac output, resulting in end-organ hypoperfusion and congestion [1]. Despite recent advances in treatment, cardiogenic shock still carries a high mortality rate of 40–60% [2]. Acute kidney injury (AKI) is a frequent form of organ injury in cardiogenic shock, affecting ~ 30% of patients surviving the initial stage [3]. Traditionally, serum creatinine and urine output are used to define AKI [4], but these markers are limited by delayed changes following kidney injury and have low sensitivity and specificity [5]. Prediction of AKI with biomarkers such as neutrophil gelatinase-associated lipocalin (NGAL) and proenkephalin (PENK) have been studied in critically ill patients [6, 7], but similar studies among cardiogenic shock patients are lacking.

PENK is a small endogenous opioid peptide, which is cleaved from the common endogenous opioid precursor, and thus can be used as a surrogate marker for the activity of the endogenous opioid system [8]. The opioid system has been shown to have depressive effects on cardiac and renal function, and has been implicated in the prognosis of myocardial infarction [9]. PENK and the opioid system have also been shown to be associated with worse outcomes both in acute [10, 11] and chronic heart failure [11]. PENK has been shown to have a strong negative correlation with eGFR measured by iohexol and is therefore a kidney biomarker of glomerular function [12]. PENK was selected as biomarker for this study as it has also been shown to predict AKI in patients with severe sepsis or shock [13].

NGAL is a member of the lipocalin protein family which is expressed in the kidney tubular structures, as well as other tissues [14]. NGAL is rapidly released in response to tubular damage [15, 16], and is one of the most extensively studied biomarkers used for AKI prediction [17].

Our aim was to assess the predictive value of P-PENK and P-NGAL for AKI within 48 h after admission and 90-day all-cause mortality in patients with cardiogenic shock.

## Methods

### Study design

The CardShock study (NCT01374867 at www.clinicaltrials.gov) was a European prospective, observational, multicenter and multinational study on cardiogenic shock. Patients were recruited between October 2010 and December 2012 from emergency departments, cardiac and intensive care units, as well as catheterization laboratories from nine tertiary hospitals in eight countries. For a detailed design and description of the study population please see Harjola et al. [18].

### Participants

Consecutive patients older than 18 years were enrolled in the study within 6 h from identification of cardiogenic shock. Written informed consent was obtained from the patient or next of kin if the patient was unable to give consent. To be included in the study, the patients needed to have (1) an acute cardiac cause for the shock, (2) systolic blood pressure < 90 mmHg (after adequate fluid challenge) for 30 min or a need for vasopressor therapy to maintain systolic blood pressure > 90 mmHg, and (3) signs of hypoperfusion (one or several of the following: altered mental status, cold periphery, oliguria < 0.5 mL/kg/h for the previous 6 h, or blood lactate > 2 mmol/L). Exclusion criteria were shock caused by ongoing hemodynamically significant arrhythmia or shock after cardiac or non-cardiac surgery. Baseline characteristics and previous medical history were recorded. Biochemical and clinical findings as well as hemodynamic parameters were documented at detection of shock and at pre-specified time points up to 48 h after inclusion. Patients were treated according to local practice and treatment and procedures were registered. Local investigators were responsible for determining the etiology of cardiogenic shock. Acute coronary syndrome etiology was defined as shock caused by myocardial infarction (with or without ST-elevation). Echocardiography was performed per protocol at study inclusion. Coronary angiography was carried out in 128 patients, of which 95 (74%) were done before study inclusion, and 24 (19%) within 12 h after study inclusion.

### Test methods

Plasma samples were collected at baseline in 178/219 patients enrolled in the CardShock study. Additionally, serial blood samples were collected at 12 h, 24 h, 48 h (all ± 3 h) and at discharge from ICU (5–10 days) (see Additional file 1: Fig. 1 for a diagram). A baseline sample and at least one additional sample within 48 h were available for 154 patients, which constitute the study population in this report. After collection, the blood sample was centrifuged, and separated plasma was immediately frozen in aliquots and stored at − 80 °C. PENK concentrations in plasma were determined using the Sphingotest^®^ penKid immunoassay (SphingoTec GmbH, Hennigsdorf, Germany). Plasma NGAL concentrations were determined with a commercially available particle-enhanced turbidimetric immunoassay (BioPorto Diagnostics A/S, Hellerup, Denmark). Creatinine, C-reactive protein (CRP), high-sensitivity troponin T, N-terminal pro-B-type natriuretic peptide (NT-proBNP), alanine aminotransferase, alkaline phosphatase, total bilirubin and cystatin C were analyzed from the plasma samples using commercially available standard kits (Abbott Laboratories, Abbott Park, IL, USA for cystatin C, Roche Diagnostics, Basel, Switzerland for all other tests) at a central accredited laboratory (ISLAB, Kuopio, Finland). Arterial blood gas analysis (including arterial pH and lactate), hemoglobin and leucocytes were analyzed by local laboratories. Estimated glomerular filtration rate (eGFR) was calculated from creatinine values using the Chronic Kidney Disease Epidemiology Collaboration equation [19]. AKI was defined and staged according to the Kidney Disease: Improving Global Outcomes (KDIGO) criteria [4]. For AKI staging at baseline, a recently described staging which includes biomarker levels was used [5]. Urine output was recorded at 6, 12, 18, and 24 h and used for urine output-based definitions of AKI. Main outcomes investigated in this study were AKI defined by an increase creatinine of more than 26,5 μmol/L within 48 h of study inclusion (AKI_crea48h_) and all-cause 90-day mortality. Assessment of AKI_crea48h_ was based on creatinine levels from baseline until 48 h, and the highest increase within this time was used for staging. AKI staging by urine output was categorized according to the lowest urine output for a time interval within the first 24 h. Subclinical AKI was defined as positive biomarker (either P-PENK or P-NGAL > cut-off) but no AKI_crea48h_. Vital status of the patients during follow-up was determined through direct contact with the patient or next of kin, or through population and hospital registers. Patients with missing plasma samples were left out of the analysis. Two patients were lost to follow-up and were left out of the survival analyses. The study was approved by local ethics committees and conducted in accordance with the Declaration of Helsinki. Two published risk scores for cardiogenic shock, CardShock risk Score [18] and IABP SHOCK II score [20] were calculated for every patient to assess whether P-PENK or P-NGAL provided additive value in risk prediction.

### Statistical analysis

Results are presented as number (*n*) and percentage (%), mean and standard deviation (SD), or median and interquartile range (IQR) as appropriate. Group comparisons were performed using Fisher’s exact test for categorical variables, and Student’s *t* test or Mann–Whitney *U* test for continuous variables, as appropriate. Associations between continuous variables were assessed using Spearman correlations. Youden index was used to select the cut-offs of P-PENK and P-NGAL used for AKI and mortality prediction. To determine variables independently associated with P-PENK and P-NGAL levels univariable general linear models were constructed using log-normalized P-PENK and P-NGAL levels as dependent variables. Differences in survival between groups were assessed comparing Kaplan–Meier survival curves using log-rank test. Cox regression models were used to assess associations of variables with AKI and mortality in uni- and multivariable models. Forward and backward selection of variables was used in multivariable Cox regression models to calculate likelihood ratios, using a significance of > 0.10 for elimination and a significance of < 0.05 for retention. The additive value of a variable in the multivariable Cox regression models was assessed using likelihood ratio test for nested models. The proportionality of hazards assumption was tested using log-minus-log plots. Hazard ratios (HR) for Cox regression analyses are shown with 95% confidence intervals (CIs). Discriminative capability of P-PENK and P-NGAL at different time points was assessed using the area under receiver-operating characteristics curve (AUC). The statistical significance of changes between different time points in P-PENK and P-NGAL levels was assessed using Wilcoxon signed ranks test. Differences in P-PENK, P-NGAL, and creatinine trajectories between different groups were assessed using linear mixed modeling. Two-sided *p* values < 0.05 were regarded as statistically significant. Data were analyzed using the SPSS statistical package, version 23 (IBM Corp, Armonk, NY, USA). STATA (StataCorp. 2019. Stata Statistical Software: Release 16. College Station, TX: StataCorp LLC) was used to calculate c-statistics for the different Cox regression models.

## Results

### Patient characteristics

Baseline characteristics of patients (*n* = 154) stratified by P-PENK and P-NGAL quartiles at baseline are presented in Table 1. The mean age of all patients in the cohort was 66 years and 26% of the patients were women. The main cause of cardiogenic shock was acute coronary syndrome, which accounted for 81% of the cases. The overall 90-day mortality was 38%. AKI was observed in 47/154 (31%) patients within 48 h of baseline. Most patients (*n* = 29) had AKI already within 12 h from baseline. Median baseline P-PENK (*n* = 152) was 105 (IQR 71–167) pmol/mL, and median baseline P-NGAL (*n* = 146) was 138 ng/mL (IQR 84–214). Patients with P-PENK > median and patients with P-NGAL > median levels had lower eGFR and hemoglobin at baseline, and had higher levels of creatinine, NT-proBNP, alkaline phosphatase and lactate. Oliguria prior to enrollment was more frequent in patients with biomarker levels > median and they had higher CardShock and IABP II Shock risk score points. Patients with PENK/NGAL > median had a higher incidence of AKI within 48 h and their mortality was higher. STARD flow diagrams of study participants are provided as Additional file 1: Fig. 2.

**Table 1:** Patient characteristics stratified by quartiles of P-PENK and P-NGAL at baseline

	All	P-PENK Q1	P-PENK Q2	P-PENK Q3	P-PENK Q4	*p*	P-NGAL Q1	P-NGAL Q2	P-NGAL Q3	P-NGAL Q4	*p*
	*N* = 154	*N* = 38	*N* = 38	*N* = 38	*N* = 38		*N* = 36	*N* = 37	*N* = 37	*N* = 36	
Age, years; (SD)	66 (12)	62 (10)	62 (15)	69 (10)	72 (11)	< 0.001	62 (13)	67 (12)	69 (11)	68 (12)	0.09
Women	40 (26%)	8 (21%)	9 (24%)	10 (26%)	13 (34%)	0.66	5 (14%)	11 (30%)	11 (30%)	10 (28%)	0.34
BMI, kg/m^2^; (SD)	27.1 (4.1)	27.5 (4.5)	27.1 (3.6)	26.6 (4.1)	26.9 (4.3)	0.84	27.3 (4.0)	25.6 (3.5)	28.0 (4.6)	27.3 (4.1)	0.09
Medical history											
Hypertension	96 (62%)	20 (53)	20 (53)	28 (64%)	26 (68%)	0.14	16 (44%)	21 (57%)	30 (81%)	25 (69%)	0.008
Coronary artery disease	51 (33%)	6 (16%)	11 (29%)	15 (40%)	19 (50%)	0.01	10 (28%)	10 (27%)	16 (43%)	15 (42%)	0.31
Previous myocardial infarction or CABG	39 (25%)	6 (16%)	9 (24%)	13 (24%)	11 (29%)	0.32	9 (25%)	7 (19%)	12 (32%)	11 (31%)	0.55
Heart failure	24 (16%)	2 (5%)	5 (13%)	8 (21%)	9 (24%)	0.13	2 (6%)	5 (14%)	6 (16%)	11 (31%)	0.03
Diabetes mellitus	43 (28%)	7 (18%)	13 (34%)	11 (29%)	12 (32%)	0.48	7 (19%)	5 (14%)	17 (46%)	13 (36%)	0.007
Renal insufficiency	17 (11%)	1 (3%)	0 (0%)	5 (13%)	11 (29%)	< 0.001	0 (0%)	1 (3%)	6 (16%)	10 (28%)	0.001
Smoking	62 (41%)	17 (45%)	22 (58%)	14 (38%)	9 (24%)	0.02	18 (50%)	15 (42%)	18 (49%)	8 (22%)	0.06
Medications in use at admission											
ACEI or ARB	63 (41%)	14 (37%)	11 (29%)	19 (50%)	18 (49%)	0.20	14 (39%)	10 (27%)	18 (49%)	17 (49%)	0.19
Diuretics	43 (28%)	5 (13%)	10 (26%)	12 (32%)	15 (41%)	0.06	6 (17%)	7 (19%)	15 (41%)	15 (43%)	0.02
Clinical presentation at baseline											
Oliguria	79 (52%)	18 (49%)	15 (40%)	19 (50%)	26 (72%)	0.04	14 (40%)	16 (43%)	20 (54%)	28 (80%)	0.003
CardShock risk score, points; mean (SD)	4.2 (1.8)	3.1 (1.4)	4.1 (1.7)	4.2 (1.8)	5.5 (1.8)	< 0.001	3.1 (1.6)	3.8 (1.6)	4.6 (1.5)	5.6 (1.7)	< 0.001
IABP II SHOCK risk score, points; mean (SD)	2.2 (1.7)	1.5 (1.3)	2.1 (1.7)	2.4 (1.8)	2.8 (1.5)	0.04	1.1 (1.2)	2.1 (1.7)	2.6 (1.8)	3.4 (1.2)	< 0.001
ACS etiology of cardiogenic shock	124 (81%)	31 (82%)	31 (82%)	32 (84%)	28 (74%)	0.77	29 (81%)	30 (81%)	32 (87%)	25 (69%)	0.33
Resuscitated	44 (29%)	10 (26%)	8 (22%)	12 (32%)	14 (37%)	0.51	7 (19%)	12 (32%)	8 (22%)	14 (39%)	0.24
Mean arterial pressure, mmHg; mean (SD)	57 (11)	59 (10)	56 (11)	56 (11)	58 (10)	0.63	57 (10)	59 (12)	57 (10)	58 (11)	0.85
LVEF, % (SD)	33 (13)	35 (15)	30 (11)	36 (13)	30 (13)	0.10	36 (14)	32 (11)	32 (11)	31 (15)	0.29
Laboratory test results at baseline											
eGFR, mL/min/1.73 m^2^; mean (SD)	65 (28)	90 (22)	70 (20)	61 (24)	39 (22)	< 0.001	89 (17)	72 (24)	59 (22)	37 (22)	< 0.001
Creatinine mg/dL; median (IQR)	1.12 (0.87–1.54)	0.76 (0.63–1.01)	1.10 (0.90–1.31)	1.18 (0.93–1.47)	1.69 (1.29–2.56)	< 0.001	0.85 (0.67–1.01)	1.00 (0.86–1.26)	1.22 (1.04–1.41)	1.93 (1.48–2.61)	< 0.001
NT-proBNP, ng/L; median (IQR)	2247 (550–8253)	1125 (242–3930)	2582 (630–8414)	1701 (251–7390)	6949 (2342–27,361)	< 0.001	1666 (371–5434)	1611 (425–5558)	2475 (678–7162)	8811 (2485–30,442)	< 0.001
hs-TnT, ng/L; median (IQR)	2275 (379–5985)	2571 (531–6643)	2439 (441–4933)	1635 (310–6820)	1857 (124–8061)	0.92	1187 (327–5336)	2862 (573–9346)	2601 (603–7689)	1597 (136–5616)	0.39
CRP, mg/L; median (IQR)	13 (4–48)	7 (4–45)	18 (5–73)	7 (3–46)	25 (9–61)	0.13	8 (4–40)	6 (3–37)	19 (5–46)	27 (9–101)	0.02
Leukocytes (10E9); mean (SD)	13.6 (5.2)	14.2 (6.1)	14.4 (4.7)	13.4 (4.5)	12.7 (5.4)	0.48	11.2 (4.2)	14.9 (5.1)	14.1 (4.6)	13.3 (6.0)	0.02
Hemoglobin, (g/dL); mean (SD)	13.0 (2.4)	13.2 (1.9)	13.2 (2.6)	13.6 (2.0)	11.9 (2.5)	0.008	12.9 (2.3)	13.5 (1.9)	13.3 (2.5)	11.8 (2.5)	0.008
Alanine aminotransferase, (IU/L); median (IQR)	42 (20–87)	29 (17–63)	43 (21–101)	42 (19–81)	54 (20–164)	0.18	24 (14–63)	46 (25–77)	43 (20–85)	83 (17–260)	0.04
Alkaline phosphatase, (IU/L); median (IQR)	61 (49–81)	55 (42–71)	64 (53–87)	62 (49–78)	65 (20–112)	0.048	53 (40–74)	62 (49–81)	70 (59–91)	64 (53–85)	0.02
Lactate, mmol/L; median (IQR)	2.6 (1.6–5.2)	1.6 (1.0–2.7)	2.5 (2.0–4.6)	2.7 (1.7–5.9)	3.9 (2.6–8.4)	< 0.001	1.8 (1.1–2.5)	2.6 (1.5–3.9)	2.6 (2.1–6.0)	5.2 (2.7–8.6)	< 0.001
P-PENK, pmol/mL; median (IQR)	99 (71–150)	54 (42–63)	81 (75–88)	120 (108–136)	211 (180–271)	< 0.001	68 (55–85)	102 (68–133)	103 (80–139)	211 (136–261)	< 0.001
P-NGAL, ng/mL; median (IQR)	138 (84–214)	78 (55–113)	132 (81–169)	146 (99–182)	287 (139–452)	< 0.001	61 (51–72)	118 (97–126)	164 (150–176)	323 (259–462)	< 0.001
AKI by 48 h	47 (31%)	5 (13%)	12 (31%)	17 (45%)	12 (30%)	0.03	4 (11%)	13 (35%)	13 (35%)	16 (44%)	0.02
90-day mortality; *N* = (%)	58 (38%)	7 (18%)	13 (35%)	15 (41%)	22 (58%)	0.005	7 (19%)	13 (35%)	17 (49%)	21 (58%)	0.005

Results shown as *n* (%) for categorical and mean (SD) or median (IQR) for continuous variables

*ACEI* angiotensin-converting enzyme inhibitor, *ACS* acute coronary syndrome, *AKI* acute kidney injury, *ARB* angiotensin receptor blocker, *BMI* body mass index, *CABG* coronary artery bypass grafting, *CRP* C-reactive protein, *eGFR* estimated glomerular filtration rate, *hs-TnT* high-sensitivity troponin T, *IQR* interquartile range, *LVEF* left ventricular ejection fraction, *NGAL* neutrophil gelatinase-associated lipocalin, *NT-proBNP* N-terminal pro-b-type natriuretic peptide, *PENK* proenkephalin, *SD* standard deviation

### Time-related changes in P-PENK and P-NGAL levels in patients with/without AKI_crea48h_ and survivors/nonsurvivors

**Fig. 1:**
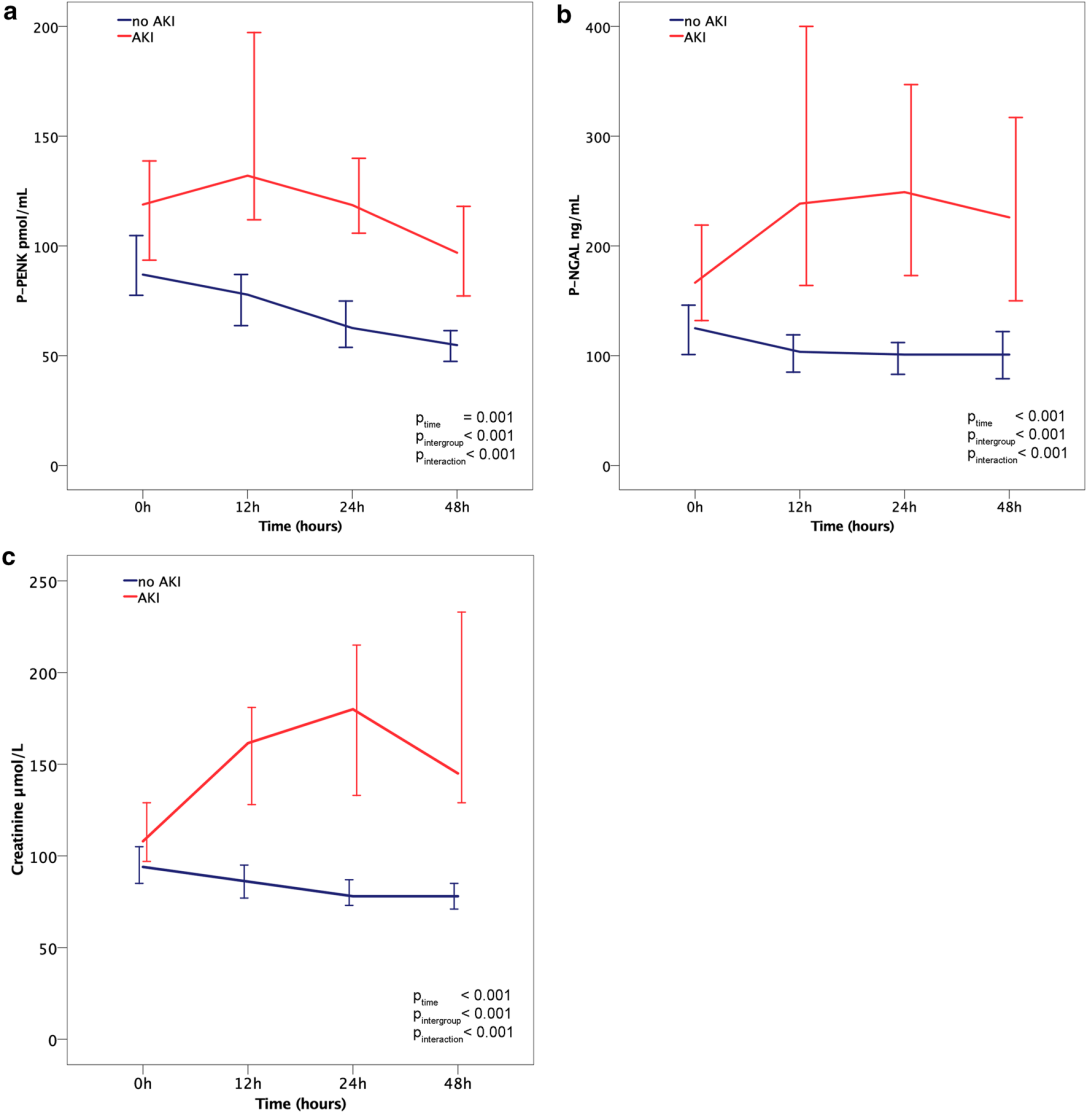
Biomarker medians at different time points separated by occurrence of AKI within 48 h. **a** P-PENK. **b** P-NGAL. **c** Creatinine. Error bars = 95% confidence interval

**Fig. 2:**
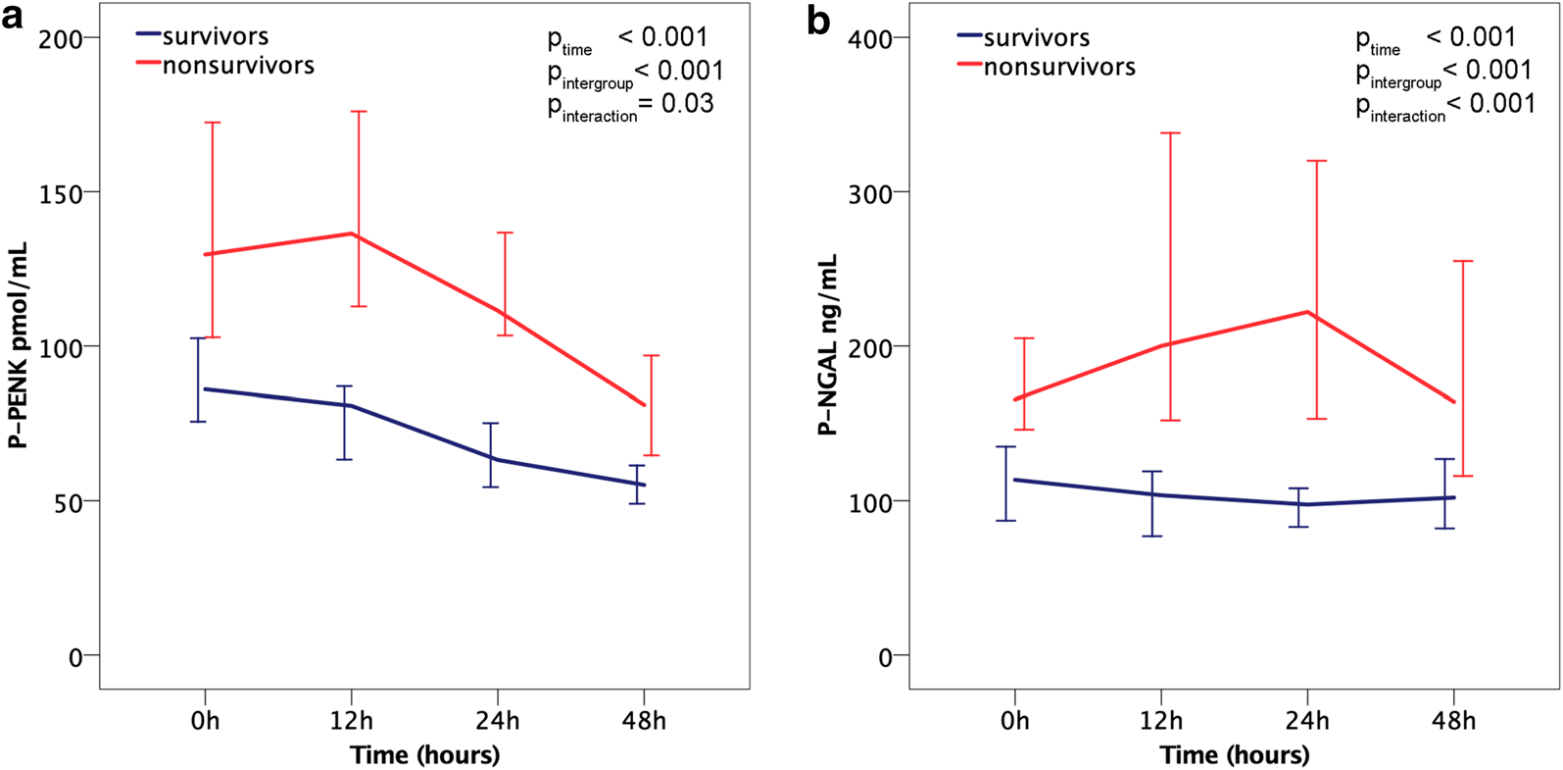
P-PENK and P-NGAL median levels at different time points separated by 90-day mortality. **a** P-PENK. **b** P-NGAL. Error bars = 95% confidence interval

Figure 1 shows the trajectories of P-PENK, P-NGAL and creatinine between baseline and 48 h in patients with and without AKI_crea48h_ and Fig. 2 shows P-PENK and P-NGAL trajectories in survivors and nonsurvivors. In nonsurvivors, the increase from baseline to 24 h was statistically significant for P-NGAL (*p* = 0.003), but not for P-PENK (*p* = 0.58). There were statistically significant differences in both P-PENK and P-NGAL levels with respect to time and group, and an interaction between the groups with time was observed.

### Association of baseline P-PENK and P-NGAL levels with AKI, renal outcomes, and interventions

**Fig. 3:**
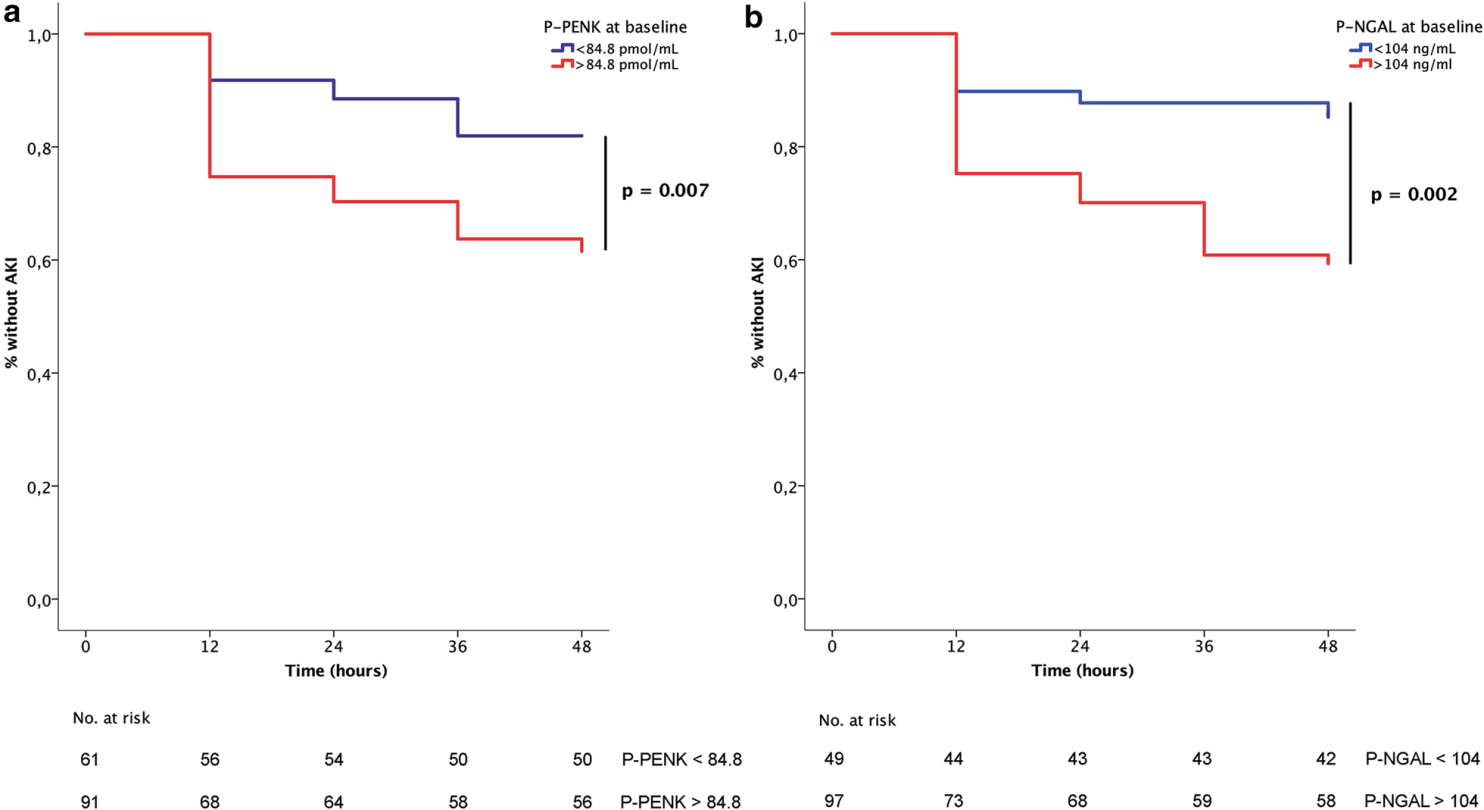
Kaplan–Meier curves for occurrence of acute kidney injury by P-PENK and P-NGAL levels at baseline. **a** Lines separated by P-PENK > 84.8 pmol/mL at baseline. **b** Lines separated by P-NGAL > 104 ng/mL at baseline

AUCs were calculated at each time point for AKI_crea48h_ and 90-day all-cause mortality to assess the discriminatory capabilities of both biomarkers (Additional file 1: Table 1). At baseline, the predictive value of P-PENK_0h_ and P-NGAL_0h_ for AKI_crea48h_ was only moderate (AUCs 0.621 and 0.664, respectively) (Additional file 1: Table 1). Compared to baseline measurements, biomarker values at later time points had better performance in prediction of AKI_crea48h_. However, since most AKI cases occurred within 12 h from baseline, P-PENK and P-NGAL measurements in time points later than baseline were not considered for AKI prediction. The optimal biomarker cut-off value at baseline for predicting AKI_crea48h_ was found to be 84.8 pmol/mL for P-PENK_0h_ and 104 ng/mL for P-NGAL_0h_. In univariable Cox regression analysis, both P-PENK_0h_ > 84.8 pmol/mL and P-NGAL_0h_ > 104 ng/mL were associated with the development of AKI (Table 2). Multivariable regression modeling identified high P-PENK_0h_ (adj HR 2.2 [95% CI 1.1–4.4] and P-NGAL_0h_ (adj HR 2.8 [95% CI 1.2–6.5]) as being independently associated with AKI_crea48h_ (Table 2). Table 3 shows renal outcomes and differences in interventions using these P-PENK_0h_ and P-NGAL_0h_ cut-offs. There were 51 (34%) patients, who fulfilled the criteria for AKI stage 1S. In patients with oliguria at baseline but without high PENK or NGAL (stage 1A) only 2/16 (13%) of patients developed AKIcrea48h, compared with 13/51 (26%) of patients in stage 1S and 33/63 (48%) in stage 1B (*p* = 0.001). Only 1/21 (5%) of patients in stage 0 developed AKIcrea48h. High P-PENK_0h_ was associated with very early AKI (< 24 h), whereas high P-NGAL_0h_ was also associated with AKI detected at 24–48 h (*p* < 0.05 for comparisons between subsets). Both high P-PENK_0h_ and high P-NGAL_0h_ were associated with AKI as defined by low urine output according to the KDIGO criteria, whereas only high P-NGAL_0h_ was associated with AKI as defined by an increase in Cystatin C. High P-NGAL_0h_ was also associated with the use of renal replacement therapy, whereas high P-PENK_0h_ was not. Figure 3 shows Kaplan–Meier curves for the development of AKI_crea48h_ for high P-PENK and high P-NGAL groups. Cross tabulation of high P-PENK and P-NGAL levels with respect to AKI_crea48h_ can be seen in Additional file 1: Table 3.

**Table 2:** Multivariable Cox regression models with P-PENK and P-NGAL at baseline for prediction of AKI_crea_ within 48 h

	Hazard ratio	95% CI	*p* value	*p* value for addition*	c-statistic with PENK/NGAL	c-statistic without PENK/NGAL
Model 1						
P-PENK > 84.8 pmol/mL at baseline	2.2	1.1–4.4	0.03	0.02	0.689	0.677
Prior use of diuretics	2.4	1.2–4.9	0.01			
Arterial pH	0.7	0.6–0.9	0.01			
Prior use of ACEI or ARB	0.4	0.2–0.8	0.01			
Model 2						
P-NGAL > 104 ng/mL at baseline	2.8	1.2–6.5	0.01	0.007	0.684	0.634
Arterial pH	0.9	0.7–1.1	0.14			

*ACEI* angiotensin-converting enzyme inhibitor, *ARB* angiotensin receptor blocker, *NGAL* neutrophil gelatinase-associated lipocalin, *PENK* proenkephalin

^*^2 log-likelihood test for additive value of P-PENK/P-NGAL in the model

**Table 3:** Differences in renal outcomes, interventions, and mortality by P-PENK and P-NGAL cut-offs at baseline

	All	PENK at 0 h < 84.8 pmol/mL	PENK at 0 h > 84.8 pmol/mL	*p* value	P-NGAL at 0 h < 104 ng/mL	P-NGAL at 0 h > 104 ng/mL	*p* value
	(*N* = 154)	(*N* = 61)	(*N* = 91)		(*N* = 49)	(*N* = 97)	
AKI staging at baseline^a^							
Stage 0	21 (14%)	21 (14%)	0 (0%)	< 0.001	17 (12%)	0 (0%)	< 0.001
Stage 1S	51 (34%)	12 (8%)	38 (26%)		13 (9%)	36 (25%)	
Stage 1A	16 (11%)	16 (11%)	0 (0%)		15 (10%)	0 (0%)	
Stage 1B	63 (42%)	11 (7%)	51 (34%)		3 (2%)	60 (42%)	
Detection of AKI by increase in creatinine after baseline							
AKI by 12 h	29 (19%)	5 (8%)	23 (25%)	0.05	5 (10%)	24 (25%)	0.006
AKI 12–24 h	6 (4%)	2 (3%)	4 (4%)		1 (2%)	5 (5%)	
AKI 24–36 h	10 (7%)	4 (7%)	6 (7%)		0 (0%)	9 (9%)	
AKI 36–48 h	2 (1%)	0 (0%)	2 (2%)		1 (2%)	1(1%)	
AKI 48 h-discharge from ICU (5–10 days)^b^	6 (4%)	2 (3%)	4 (4%)	0.73	3 (6%)	3 (3%)	0.38
AKI CysC	49 (33%)	17 (29%)	32 (36%)	0.32	9 (18%)	40 (42%)	0.005
AKI severity by urine output after baseline							
No	75 (50%)	37 (61%)	38 (42%)	0.04	31 (63%)	42 (44%)	0.02
Stage 1	33 (22%)	13 (21%)	20 (22%)		11 (22%)	19 (20%)	
Stage 2 or 3	43 (29%)	11 (18%)	32 (36%)		7 (14%)	35 (37%)	
AKI severity within 48 h of baseline by creatinine (RRT excluded from staging)				0.008			0.005
No AKI	106 (70%)	50 (82%)	56 (61%)		42 (86%)	58 (60%)	
Stage 1	27 (18%)	9 (15%)	18 (20%)		5 (10%)	22 (23%)	
Stage 2 or 3 (RRT excluded)	19 (12%)	2 (3%)	17 (19%)		2 (4%)	17 (17%)	
History of renal insufficiency	17 (11%)	1 (2%)	16 (18%)	0.003	0 (0%)	17 (18%)	0.002
Interventions							
Renal replacement therapy	22 (14%)	7 (12%)	15 (17%)	0.49	3 (6%)	19 (20%)	0.048
Ultrafiltration	11 (8%)	2(4%)	9 (11%)	0.20	2 (5%)	9 (11%)	0.33
Hemodialysis	7 (5%)	0 (0%)	7 (9%)	0.04	0 (0%)	7 (8%)	0.10
Use of vasopressors	126 (83%)	50 (82%)	76 (84%)	0.83	41 (84%)	81 (84%)	0.99
Use of adrenaline	21 (14%)	7 (12%)	14 (15%)	0.63	2 (4%)	18 (19%)	0.02
Use of noradrenaline	114 (75%)	44 (72%)	70 (77%)	0.57	39 (80%)	73 (75%)	0.68
Use of dobutamine	25 (16%)	11 (18%)	14 (15%)	0.82	5 (10%)	18 (19%)	0.23
History of renal insufficiency	17 (11%)	1 (2%)	16 (18%)	0.003	0 (0%)	17 (18%)	0.002
Coronary angiography	128 (83%)	55 (90%)	71 (78%)	0.08	45 (92%)	75 (77%)	0.04
Amount of contrast used; mL (SD)	189 (104)	192 (106)	186 (105)	0.72	183 (113)	197 (102)	0.29
Amount of contrast used per eGFR; mL/eGFR (SD)	3.4 (2.4)	2.5 (1.6)	4.1 (3.0)	0.005	2.3 (1.8)	4.2 (3.6)	< 0.001
IABP use	86 (57%)	39 (64%)	47 (52%)	0.18	27 (55%)	56 (58%)	0.86
Intubated	93 (62%)	36 (60%)	57 (63%)	0.73	28 (57%)	61 (64%)	0.47
LVAD or ECMO	6 (4%)	2 (3%)	4 (4%)	0.99	1 (2%)	4 (4%)	0.66
90-day mortality	58 (38%)	15 (25%)	42 (47%)	0.006	9 (18%)	49 (52%)	< 0.001

Results shown as *n* (%) for categorical and mean (SD) or median (IQR) for continuous variables

*AKI* acute kidney injury, *CysC* cystatin C, *ECMO* extracorporeal membrane oxygenation, *eGFR* estimated glomerular filtration rate, *IABP* intra-aortic balloon pump, *ICU* intensive care unit, *IQR* interquartile range, *LVAD* left ventricular assist device, *NGAL* neutrophil gelatinase-associated lipocalin, *PENK* proenkephalin, *RRT* renal replacement therapy, *SD* standard deviation

^a^AKI staging on admission based on urine output and biomarkers, as data on creatinine prior to baseline were unavailable

^b^KDIGO definition of increase in serum creatinine to 1.5 times baseline or more

### Association of baseline levels of P-PENK and P-NGAL with 90-day mortality

**Fig. 4:**
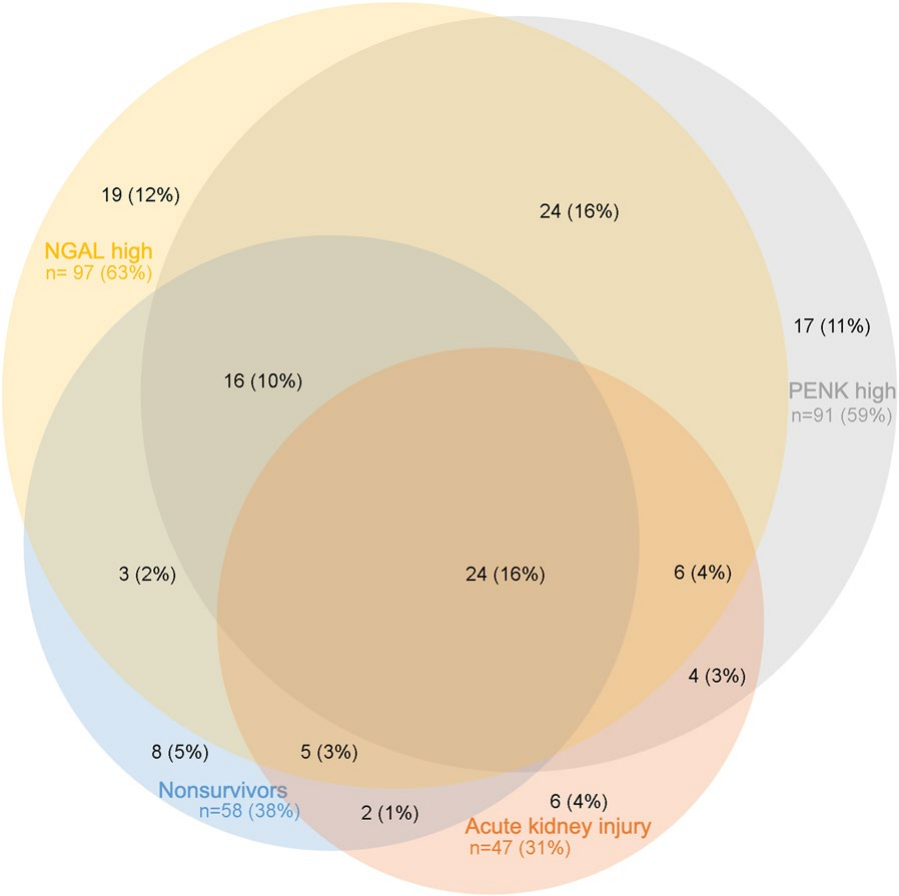
Venn diagram of P-PENK, P-NGAL at baseline, acute kidney injury and 90-day mortality. PENK high = P-PENK < 84.8 pmol/mL at baseline. P-NGAL high = P-NGAL > 104 ng/mL at baseline. For illustrative purposes. Areas not proportional, unable to show all overlaps

**Fig. 5:**
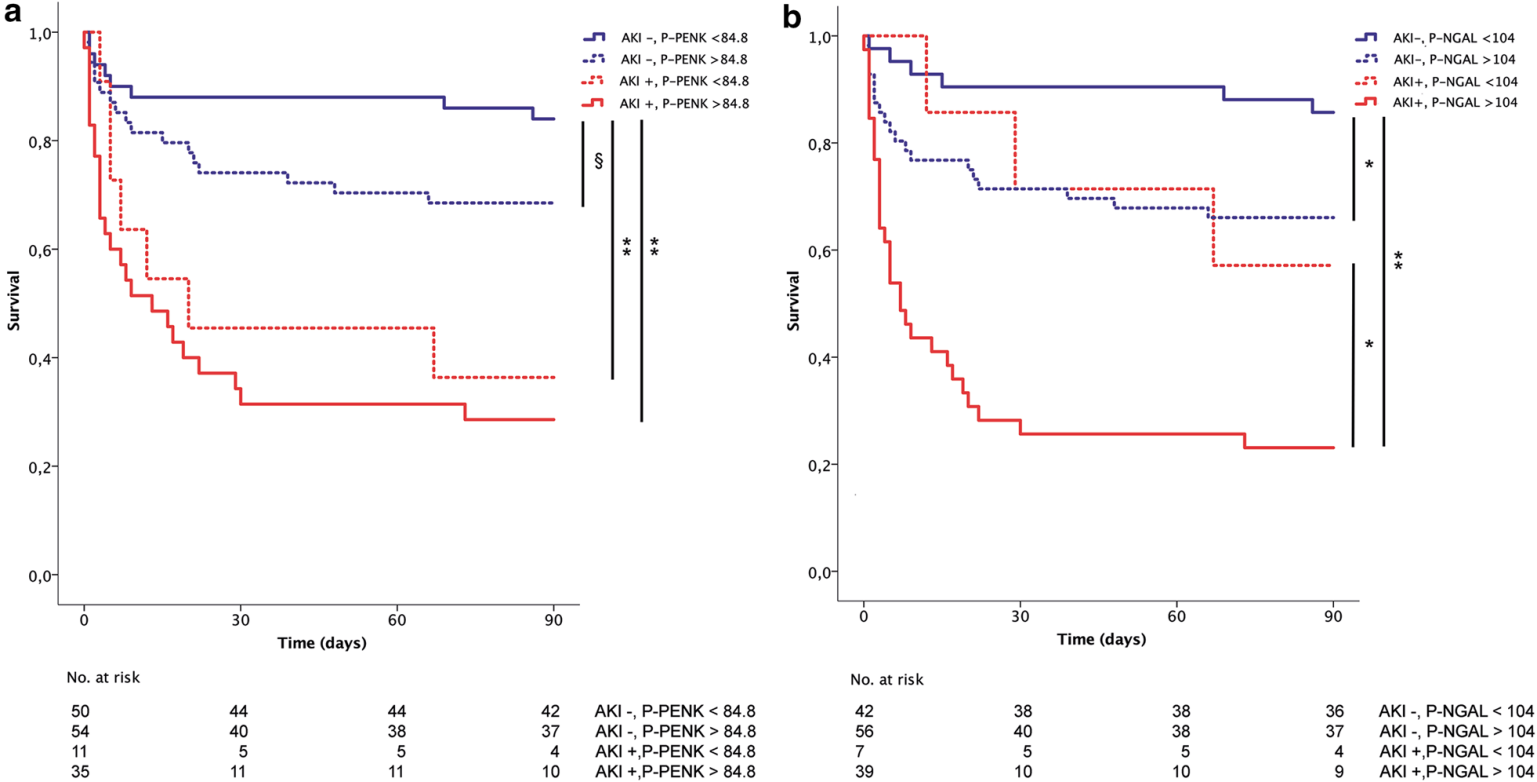
Kaplan–Meier survival curves for patients with and without AKI separated by P-PENK and P-NGAL levels. **a** P-PENK higher or lower than 84.8 pmol/mL at baseline. **b** P-NGAL higher or lower than 104 ng/mL at baseline. §*p* = 0.07, *p < 0.05, **p < 0.001. *AKI* acute kidney injury

The relationships between AKI, 90-day mortality and high P-PENK_0h_ and/or P-NGAL_0h_ are depicted as a Venn diagram in Fig. 4. 90-day mortality differed significantly between AKI stages at admission (7% for stage 0, 19% for stage 1S, 5% for stage 1A and 68% for stage 1B, *p* < 0.001). Both P-PENK_0h_ > 84.8 pmol/mL and P-NGAL_0h_ > 104 ng/mL were associated with higher 90-day mortality (Table 3). Figure 5 shows the survival curves for patients with and without AKI_crea48h_ separated by baseline P-PENK (panel A) and baseline P-NGAL (panel B) higher (subclinical AKI) or lower than the optimal cut-off for AKI prediction (84.8 pmol/mL for P-PENK_0h_ and 104 ng/mL for P-NGAL_0h_). Using the cut-offs predictive of AKI, P-NGAL_0h_ > 104 ng/mL was able to further stratify patients with and without AKI_crea48h_ further into high or low mortality risk groups (Fig. 5).

### Utility of P-PENK and P-NGAL at later time points for mortality risk stratification in cardiogenic shock

Although high levels of both P-PENK and P-NGAL were associated with higher mortality at all time points examined, the AUC for mortality was highest for both biomarkers measured at 24 h (Additional file 1: Table 2). The AUCs for 90-day mortality for CardShock risk score and IABP SHOCK II risk score at 24 h were 0.778 and 0.707.

The optimal cut-off for mortality was 105.7 pmol/mL for P-PENK_24h_, and 151 ng/mL for P-NGAL_24h_. Survival curves using these cut-offs showed that for patients with P-PENK_24h_ > 105.7 pmol/mL 90-day mortality was 68.2% compared with 17.4% for patients with P-PENK_24h_ < 105.7 pmol/mL. Similarly, 90-day mortality for patients with P-NGAL_24h_ > 151 ng/mL was 63.5% compared with 17.7% for patients with P-NGAL_24h_ < 151 ng/mL (*p* < 0.001 for all, Additional file 1: Fig. 2).

In univariable Cox regression, both P-PENK_24h_ > 105.7 pmol/mL and P-NGAL_24h_ > 151 ng/mL were strongly associated with mortality (Table 4). In multivariable analysis, the associations of both P-PENK_24h_ > 105.7 pmol/mL and P-NGAL_24h_ > 151 ng/mL with higher mortality were found to be independent of both CardShock risk score and IABP II Shock score. Further adjustment of the multivariable models with AKI_crea48h_ did not change the independent association of P-PENK_24h_ with 90-day all-cause mortality, whereas P-NGAL_24h_ was no longer significantly associated with 90-day mortality after adjusting for both CardShock risk score and AKI_crea48h_. Cross tabulation of P-PENK_24h_ and P-NGAL_24h_ with respect to 90-day mortality can be seen in Additional file 1: Table 3.

**Table 4:** Hazard ratios for multivariable models of 90-day mortality for P-PENK_24h_ > 105.7 pmol/mL and P-NGAL_24h_ > 151 ng/mL

	Hazard ratio	95% CI	*p* value*	c-statistic	c-statistic without PENK/NGAL
P-PENK_24h_ > 105.7 pmol/mL at 24 h					
Univariable	5.8	3.1–10.7	< 0.001	0.705	
Adjusted model 1 (CardShock risk score)	4.5	2.3–8.7	< 0.001	0.778	0.726
Adjusted model 2 (IABP II SHOCK score)	4.2	2.0–9.0	< 0.001	0.750	0.702
Adjusted model 3 (AKIcrea48h)	3.5	1.7–6.9	< 0.001	0.750	0.671
Adjusted model 4 (CardShock risk score + AKIcrea48h)	2.7	1.3–5.6	0.01	0.800	0.781
Adjusted model 5 (IABP II SHOCK score + AKIcrea48h)	2.7	1.2–5.9	0.01	0.773	0.749
P-NGAL_24h_ > 151 ng/mL at 24 h					
Univariable	5.2	2.8–9.8	< 0.001	0.632	
Adjusted model 1 (CardShock risk score)	3.4	1.7–6.8	0.001	0.733	0.726
Adjusted model 2 (IABP II SHOCK score)	4.2	2.0–8.9	< 0.001	0.724	0.702
Adjusted model 3 (AKIcrea48h)	3.2	1.6–6.5	0.001	0.716	0.671
Adjusted model 4 (CardShock risk score + AKIcrea48h)	2.0	0.9–4.2	0.07	0.781	0.781
Adjusted model 5 (IABP II SHOCK score + AKIcrea48h)	2.5	1.1–5.9	0.03	0.755	0.749

*AKIcrea48h* acute kidney injury by creatine increase within 48 h, *CI* confidence interval, *NGAL* neutrophil gelatinase-associated lipocalin, *PENK* proenkephalin

^*^2 log-likelihood test for additive value of P-PENK/P-NGAL in the model

### Determinants of P-PENK and P-NGAL in cardiogenic shock

Results from a Spearman correlation analysis with baseline variables and levels of P-PENK and P-NGAL are shown in Additional file 1: Table 2. Markers of renal function had moderate to strong correlations with both P-PENK and P-NGAL at baseline. P-PENK and P-NGAL were also intercorrelated. General linear model analysis showed that the biomarkers had strongest associations with eGFR (F-statistic 82.8 for P-PENK and 84.9 for P-NGAL; *p* < 0.001 for both) and log-normalized lactate levels at baseline (F-statistic 5.0; *p* = 0.03 for P-PENK and 16.4; *p* < 0.001 for P-NGAL). In addition, body mass index (F-statistic 6.0, *p* = 0.02) and alkaline phosphatase (F-statistic 5.0, *p* = 0.03 were independent associates of P-NGAL levels. eGFR alone accounted for 36% of the observed variance of P-PENK and 39% of P-NGAL variance.

## Discussion

This multicenter cohort study is the first to investigate P-PENK and P-NGAL in cardiogenic shock of various etiologies. We describe the early kinetics of P-PENK and P-NGAL and their associations with markers of kidney function, AKI and outcomes. The main findings can be summarized as follows. Firstly, both P-PENK and P-NGAL at baseline correlated with markers of kidney function. Secondly, high baseline levels of both studied markers were associated with the development of AKI within 48 h. Thirdly, subclinical AKI at baseline was associated with increased mortality both in patients with oliguria before study enrollment as well as in patients without AKI_crea48h_. Finally, although high levels of P-PENK and P-NGAL were associated with worse outcomes at all studied time points, P-PENK and P-NGAL levels at 24 h had the best discriminatory capabilities on survival. They were also associated with mortality independently of the development of AKI and two separate risk scores designed to assess the prognosis of patients in cardiogenic shock.

### P-PENK and P-NGAL as predictors of AKI

Elevated levels of P-PENK and P-NGAL were both associated with the development of AKI_crea48h_ and had comparable discriminatory properties in AUC analysis. Interestingly, we found that AKI_crea48h_ occurred almost solely if oliguria (< 0.5 mL/kg/h for > 6 h) before study inclusion was combined with subclinical AKI at baseline. P-NGAL has been shown to predict AKI after cardiac surgery [21], in critically ill children [22] and adults [23] with suggested cut-offs ranging from 100 to 270 nmol/mL [24]. In a recent meta-analysis comparing plasma/serum NGAL, urine NGAL and serum cystatin C, plasma/serum NGAL was found to be the earliest marker predicting contrast-induced nephropathy [25]. Of the two markers investigated in the present study, only P-NGAL was associated with renal replacement therapy and AKI assessed by changes in cystatin C plasma concentrations, suggesting P-NGAL may be a more robust indicator of AKI and other renal outcomes. For most patients who developed AKI, it occurred very early, which makes it most likely related to the acute state of cardiogenic shock and not secondary to other events in-hospital. Thus, based on our findings, the ability to predict the occurrence and severity of AKI using biomarkers measured at presentation opens a narrow window of opportunity for targeted interventions.

### Biomarker trajectories in AKI and nonsurvivors

Overall, levels of P-PENK and P-NGAL decreased from 0 to 48 h. In nonsurvivors and patients who developed AKI within 48 h the levels were higher at all time points for both P-PENK and P-NGAL compared to survivors and patients without AKI. While the absolute levels differed, the trajectories of P-PENK over time were similar in survivors and nonsurvivors. In contrast, for P-NGAL, the levels were increasing up to 48 h in nonsurvivors, but were stable or declined in survivors. These findings most likely represent differences in the production and kinetics of the two biomarkers. In a mouse renal ischemia–reperfusion injury model, P-NGAL expression has been shown to be upregulated in the proximal kidney tubular cells early in response to renal ischemia with a peak at 12 h post-ischemia [26], which could explain the initial increase observed for P-NGAL in nonsurvivors and patients who developed AKI and also had higher levels of lactate. P-PENK, on the other hand, is a marker of the endogenous opioid system activity, which could be higher in those with more severe clinical presentation, but be more stable over time. P-PENK could also cause a deterioration of renal function through cardiodepression [27], with high initial levels causing a more marked decline in kidney function. These pathophysiological mechanisms may be seen to be in concordance with the results of our study that patients with high levels of either P-PENK or P-NGAL were at significantly higher risk for mortality and for developing AKI within 48 h.

### Association of P-PENK and P-NGAL with mortality

Both P-PENK and P-NGAL showed good discriminatory capabilities for 90-day mortality at all time points up to 48 h, with the highest AUCs for both occurring at 24 h. High levels of both P-PENK_24h_ and P-NGAL_24h_ were associated independently with 90-day mortality even after adjusting for the CardShock risk score, IABP II SHOCK risk score and AKI. In fact, the AUCs for both P-PENK_24h_ and P-NGAL_24h_ were similar to those of the risk scores alone. The independent association of P-PENK_24h_ and P-NGAL_24h_ with 90-day mortality suggests that they are not just markers for AKI, but may also contribute to other unidentified causes of increased mortality in the CardShock study population that are not included in the relevant risk scores. A recent study in critically ill patients suggests that high levels of P-PENK in patients without AKI may be a marker of subclinical AKI, which was associated with a risk of death close to patients with AKI [28]. Similarly in our study, patients with subclinical AKI (elevated biomarkers at baseline but without AKI_crea48h_) had higher 90-day mortality. Interestingly, P-NGAL was also able to stratify patients with AKI_crea48h_ into high or low mortality risk groups. It could be hypothesized that this might be due to less severe AKI, where despite an increase in creatinine the renal injury is smaller. Using the recently described AKI staging using biomarkers on admission [5], we were also able to show that low urine output (< 0.5 mL/kg/h for > 6 h) before study inclusion was associated with worse outcomes only if combined with high baseline levels of P-PENK or P-NGAL.

Elevated P-NGAL may not be specific for acute kidney injury, as NGAL is also expressed at low levels in several human tissues, including lung, stomach, and colon, as well as neutrophils [14]. Serum NGAL levels have been shown to increase also with infection, inflammation and ischemia [29]. The association of NGAL with inflammation and ischemia might be a drawback in acute kidney injury prediction, but might help explain the high discriminatory capability in 90-day mortality in cardiogenic shock, where systemic inflammatory response syndrome and end-organ ischemia play an important role. P-PENK and P-NGAL could be useful in assessing mortality risk of cardiogenic shock patients at later time points for which few risk markers have so far been assessed [30]. However, although this study offers new insight to prediction of AKI and mortality in cardiogenic shock populations, overall data on biomarkers in this area are still very limited and more research is warranted. The results of our study should be validated in another cohort of cardiogenic shock patients to confirm our findings.

### Study limitations

We did not have plasma samples available for all the CardShock study participants, and also were unable to measure P-NGAL for some of the patients in this study. However, this is one of the largest cohorts of biomarker studies in patients with cardiogenic shock and despite the general challenges with serial sampling in acute cardiac care, we consider the available samples to be representative of the cohort. One of the study limitations is the lack of data on creatinine prior to study inclusion. On the other hand, the European Renal Best Practice position statement recommends using baseline creatinine values instead of historical values or back-calculated value based on an assumed GFR of 75 mL/min/1.73 m^2^ [31]. Although adjustments were made for several variables in multivariable analyses, there may have been other confounding factors we were unable to account for leading to an overestimation of the independent association of P-PENK and P-NGAL with AKI and mortality.

## Conclusions

Our study shows that in cardiogenic shock, P-PENK and P-NGAL levels differed between patients who did and did not develop AKI within 48 h as well as between 90-day survivors and nonsurvivors. High levels of both P-PENK and P-NGAL at presentation were associated with AKI within 48 h. Subclinical AKI at baseline was associated with increased mortality both in patients with oliguria before study enrollment as well as in patients without AKI_crea48h_. Adjusting for AKI and two risk scores validated for cardiogenic shock patient populations, high levels of P-PENK and P-NGAL at 24 h were still independently associated with higher 90-day all-cause mortality. P-PENK and P-NGAL seem useful biomarkers in the early prediction of outcomes in cardiogenic shock populations, and may have role in the prediction of AKI.

## Supplementary Information

**Additional file 1** : **Table S1**. Areas under the receiver-operating characteristic curve for acute kidney injury occurring within 48 hours and 90-day all-cause mortality for P-PENK and P-NGAL at different time points. **Table S2**. Spearman correlations between P-PENK and P-NGAL and other variables at baseline. **Table S3**. Cross tabulation of index test results. **Figure S1**. Diagram of study sampling times. **Figure S2**. STARD flow diagram of study participants. A: AKI B: 90-day mortality. **Figure S3**. Kaplan–Meier survival curves stratified by a) P-PENK >105.7 pmol/mL at 24 hours and b) P-NGAL >151 ng/mL at 24 hours.

## Data Availability

The datasets used and analyzed during the current study are available from the corresponding author on reasonable request.

## Abbreviations

AKI: Acute kidney injury
AUC: Area under the receiver-operating characteristics curve
CRP: C-reactive protein
eGFR: Estimated glomerular filtration fraction
HR: Hazard ratio
IABP SHOCK II: Intra-aortic balloon counterpulsation in acute myocardial infarction complicated by cardiogenic shock trial
IQR: Interquartile range
KDIGO: Kidney disease: improving global outcomes
NGAL: Neutrophil gelatinase-associated lipocalin
NT-proBNP: N-terminal Pro-b-type natriuretic peptide
PENK: Proenkephalin
SD: Standard deviation
