# Supplementary material for: Predictive value of plasma proenkephalin and neutrophil gelatinase-associated lipocalin in acute kidney injury and mortality in cardiogenic shock
Source: Ann Intensive Care. 2021 Feb 5;11:25. doi: 10.1186/s13613-021-00814-8 (PMC7865050; doi:10.1186/s13613-021-00814-8)

**Supplementary Table 1. Areas under the receiver-operating characteristic curve for acute kidney injury occurring within 48 hours and 90-day all-cause mortality for P-PENK and P-NGAL at different time points.**

|  | AKI |  |  |  |
| --- | --- | --- | --- | --- |
|  | 0h | 12h | 24h | 48h |
|  | N=152 | N=148 | N=138 | N=122 |
| P-PENK | 0.621 | 0.743 | 0.803 | 0.779 |
|  | N=146 | N=102 | N=133 | N=116 |
| P-NGAL | 0.664 | 0.822 | 0.810 | 0.804 |
|  |  |  |  |  |
|  | 90-day mortality | |  |  |
|  | 0h | 12h | 24h | 48h |
|  | N=152 | N=148 | N=138 | N=122 |
| P-PENK | 0.684 | 0.736 | 0.756 | 0.687 |
|  | N=146 | N=102 | N=133 | N=116 |
| P-NGAL | 0.701 | 0.776 | 0.783 | 0.711 |

N=number of patients with samples available

**Supplementary Table 2. Spearman correlations between P-PENK and P-NGAL and other variables at baseline.**

| \| P-PENK \|  \|  \| P-NGAL \|  \|  \| \| --- \| --- \| --- \| --- \| --- \| --- \| \|  \| r(s) \| p \|  \| r(s) \| p \| \| eGFR \| -0.657 \| <0.001 \| eGFR \| -0.706 \| <0.001 \| \| CysC \| 0.650 \| <0.001 \| Crea \| 0.692 \| <0.001 \| \| P-NGAL \| 0.629 \| <0.001 \| CysC \| 0.683 \| <0.001 \| \| Crea \| 0.619 \| <0.001 \| P-PENK \| 0.629 \| <0.001 \| \| Lactate \| 0.421 \| <0.001 \| Lactate \| 0.513 \| <0.001 \| \| Age \| 0.365 \| <0.001 \| NT-proBNP \| 0.332 \| <0.001 \| \| NT-proBNP \| 0.296 \| <0.001 \| Age \| 0.261 \| 0.001 \| \| AP \| 0.173 \| 0.03 \| CRP \| 0.238 \| 0.004 \| \|  \|  \|  \| AP \| 0.229 \| 0.006 \| \|  \|  \|  \| ALT \| 0.222 \| 0.007 \| |  |  |  |  |  |
| --- | --- | --- | --- | --- | --- | --- | --- | --- | --- | --- | --- | --- | --- | --- | --- | --- | --- | --- | --- | --- | --- | --- | --- | --- | --- | --- | --- | --- | --- | --- | --- | --- | --- | --- | --- | --- | --- | --- | --- | --- | --- | --- | --- | --- | --- | --- | --- | --- | --- | --- | --- | --- | --- | --- | --- | --- | --- | --- | --- | --- | --- | --- | --- | --- | --- | --- | --- | --- | --- | --- | --- | --- | --- | --- | --- | --- | --- |

**Supplementary Table 3. Cross tabulation of index test results**

| \|  \| no AKIcrea in 48hrs \| AKIcrea in 48 hrs \| \|  \|  \| no AKIcrea in 48hrs \| AKIcrea in 48 hrs \| \| \| --- \| --- \| --- \| --- \| --- \| --- \| --- \| --- \| --- \| \| PENK0h <84.8 \| 50 \| 11 \| 61 \|  \| NGAL0h <104 \| 42 \| 7 \| 49 \| \| PENK0h >84.8 \| 56 \| 35 \| 91 \|  \| NGAL0h >104 \| 58 \| 39 \| 97 \| \|  \| 106 \| 46 \|  \|  \|  \| 100 \| 46 \|  \| \|  \|  \|  \|  \|  \|  \|  \|  \|  \| \|  \| 90-day survivors \| 90-day nonsurvivors \| \|  \|  \| 90-day survivors \| 90-day nonsurvivors \| \| \| PENK24h <105.7 \| 76 \| 16 \| 92 \|  \| NGAL24h <151 \| 65 \| 14 \| 79 \| \| PENK24h >105.7 \| 14 \| 30 \| 44 \|  \| NGAL24h >151 \| 19 \| 33 \| 52 \| \|  \| 90 \| 46 \|  \|  \|  \| 84 \| 47 \|  \| |  |  |  |  |  |  |  |
| --- | --- | --- | --- | --- | --- | --- | --- | --- | --- | --- | --- | --- | --- | --- | --- | --- | --- | --- | --- | --- | --- | --- | --- | --- | --- | --- | --- | --- | --- | --- | --- | --- | --- | --- | --- | --- | --- | --- | --- | --- | --- | --- | --- | --- | --- | --- | --- | --- | --- | --- | --- | --- | --- | --- | --- | --- | --- | --- | --- | --- | --- | --- | --- | --- | --- | --- | --- | --- | --- | --- | --- | --- | --- | --- | --- | --- | --- | --- | --- | --- | --- | --- | --- | --- | --- | --- | --- | --- |

**Supplementary Figure 1. Diagram of study sampling times (arrows)**

**Supplementary Figure 2. STARD flow diagram of study participants. A: AKI B: 90-day mortality**

**A B**

**Supplementary Figure 2. Kaplan-Meier survival curves stratified by a) P-PENK >105.7 pmol/mL at 24 hours and b) P-NGAL >151ng/mL at 24 hours.**


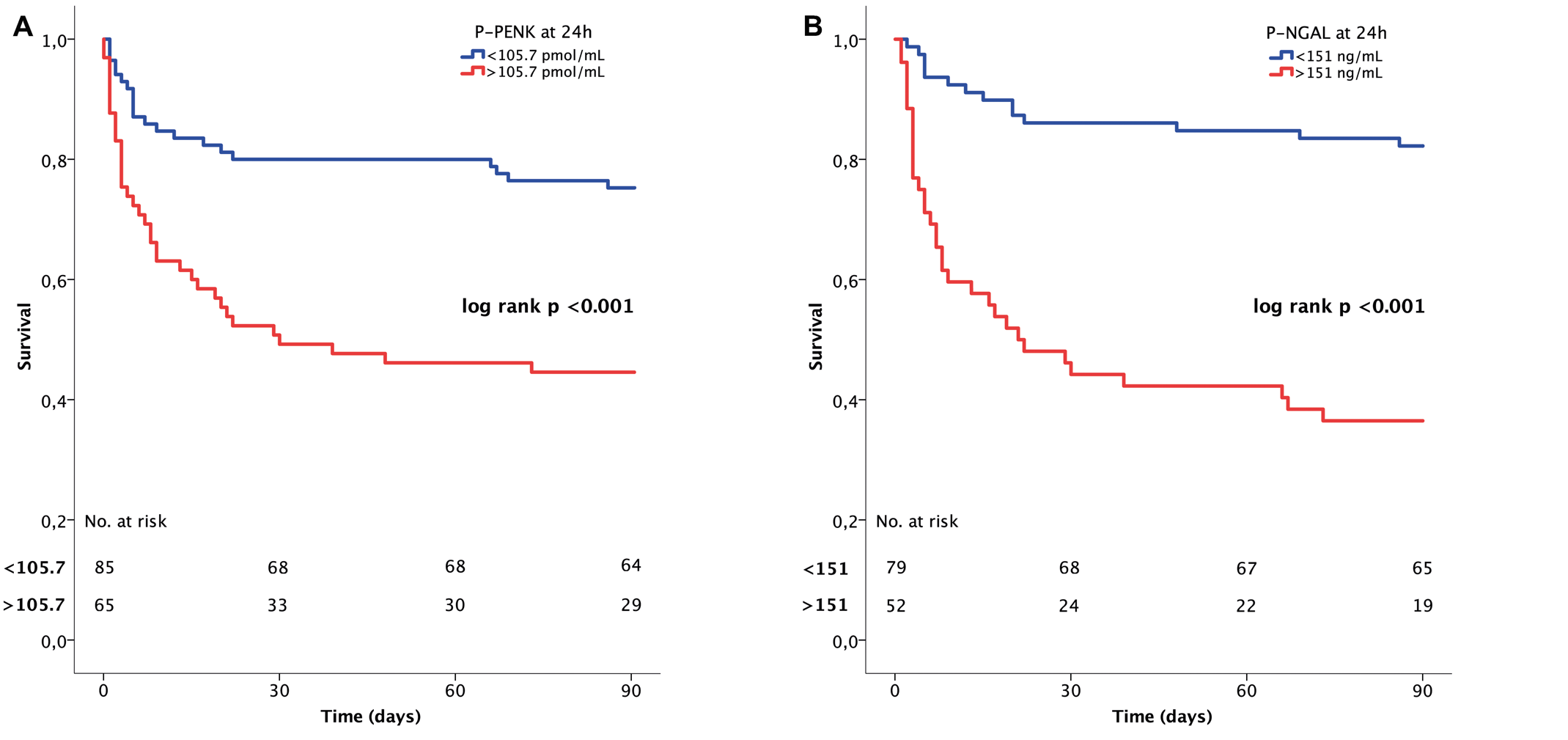

Supplement: Supplementary file 1 — Additional file 1 : Table S1. Areas under the receiver-operating characteristic curve for acute kidney injury occurring within 48 hours and 90-day all-cause mortality for P-PENK and P-NGAL at different time points. Table S2. Spearman correlations between P-PENK and P-NGAL and other variables at baseline. Table S3. Cross tabulation of index test results. Figure S1. Diagram of study sampling times. Figure S2. STARD flow diagram of study participants. A: AKI B: 90-day mortality. Figure S3. Kaplan–Meier survival curves stratified by a) P-PENK >105.7 pmol/mL at 24 hours and b) P-NGAL >151 ng/mL at 24 hours. [file 13613_2021_814_MOESM1_ESM.docx]
